# 5,7-Di-*N*-acetyl-8-epiacinetaminic acid: A new non-2-ulosonic acid found in the K73 capsule produced by an *Acinetobacter baumannii* isolate from Singapore

**DOI:** 10.1038/s41598-017-11166-4

**Published:** 2017-09-12

**Authors:** Johanna J. Kenyon, Anna Notaro, Li Yang Hsu, Cristina De Castro, Ruth M. Hall

**Affiliations:** 1 School of Life and Environmental Sciences, The University of Sydney, Sydney, Australia; 2 Institute of Health and Biomedical Innovation, Queensland University of Technology, Brisbane, Australia; 3 Department of Chemical Sciences, University of Napoli, Naples, Italy; 4 Saw Swee Hock School of Public Health, National University of Singapore, Singapore, Singapore; 5 Department of Infectious Diseases, Tan Tock Seng Hospital, Singapore, Singapore; 6 Department of Agricultural Sciences, University of Napoli, Naples, Italy

## Abstract

Nonulosonic acids are found in the surface polysaccharides of many bacterial species and are often implicated in pathogenesis. Here, the structure of a novel 5,7-diacetamido-3,5,7,9-tetradeoxynon-2-ulosonic acid recovered from the capsular polysaccharide of a multiply antibiotic resistant *Acinetobacter baumannii* isolate was determined. The isolate carries a sugar synthesis module that differs by only a single gene from the module for the synthesis of 5,7-diacetamido-3,5,7,9-tetradeoxy-L-*glycero*-L-*altro*-non-2-ulosonic acid or 5,7-di-*N*-acetylacinetaminic acid, recently discovered in the capsule of another *A. baumannii* isolate. The new monosaccharide is the C8-epimer of acinetaminic acid (8eAci; 5,7-diacetamido-3,5,7,9-tetradeoxy-D-*glycero*-L-*altro*-non-2-ulosonic acid) and the C7-epimer of legionaminic acid. This monosaccharide had not previously been detected in a biological sample but had been synthesized chemically.

## Introduction

**Figure 1:**
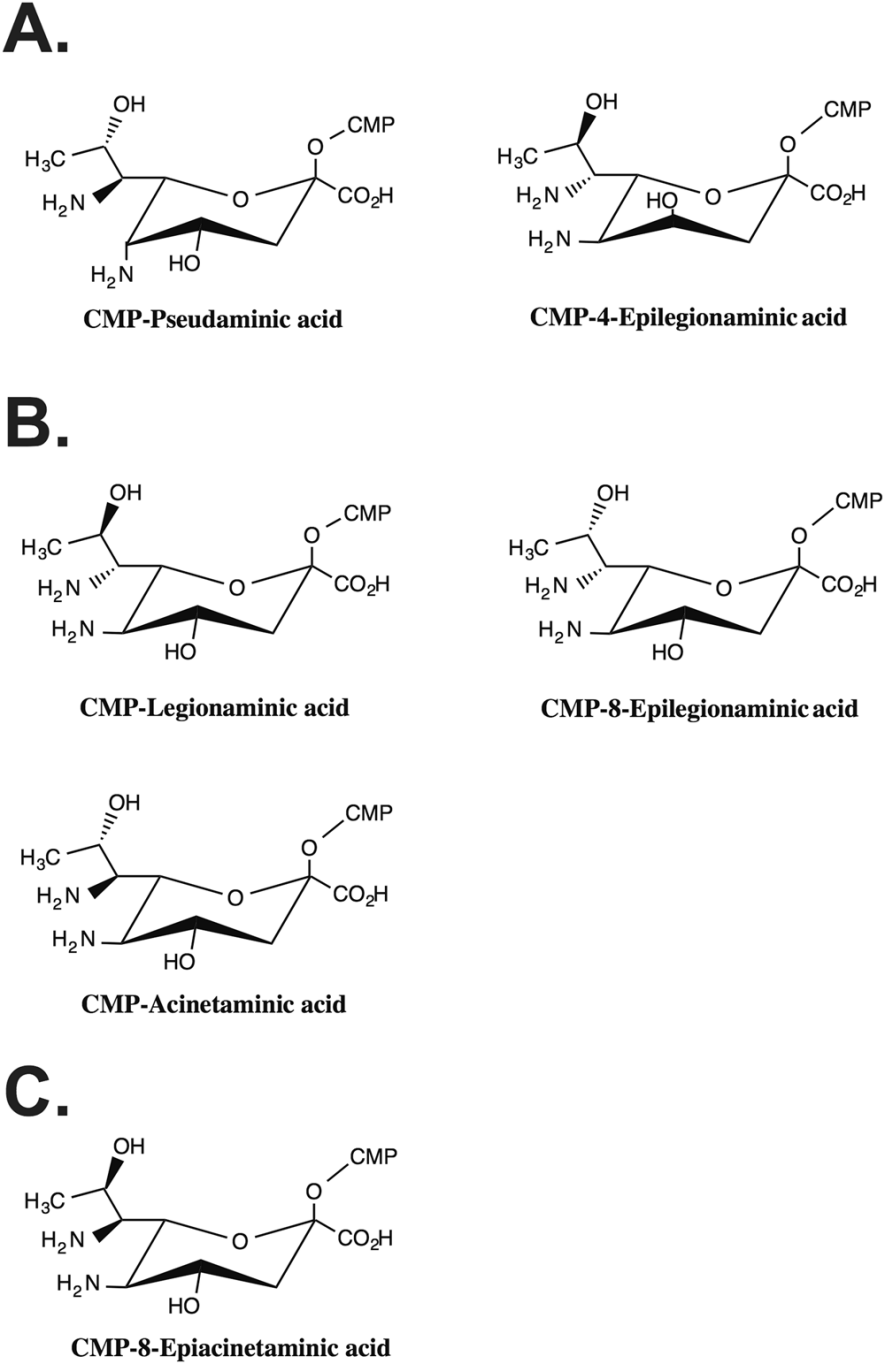
Structures of naturally occurring 5,7-diamino-3,5,7,9-tetradeoxynon-2-ulosonic acids. (**A**) Structures of 5,7-diamino-3,5,7,9-tetradeoxynon-2-ulosonic acids discovered prior to 2017 synthesised via pathways that do not involve *lga* biosynthesis genes. (**B**) Structures of 5,7-diamino-3,5,7,9-tetradeoxynon-2-ulosonic acids discovered prior to 2017 requiring *lga* biosynthesis genes. (**C**) Structure of 8-epiacinetaminic acid (this study).

The nonulosonate superfamily of monosaccharides includes various acetyl and acyl derivatives of nine-carbon acidic sugars such as neuraminic acid and sialic acid, which have important implications for human health^1^. This family includes a subclass made up of 5,7-diamino-3,5,7,9-tetradeoxynon-2-ulosonic acids that is exclusively found in bacteria^2^. Currently this subclass includes five natural isomers found in biological samples, some of which have been shown to be associated with the virulence of bacterial pathogens. Pseudaminic acid (Pse; L-*glycero*-L*-manno* isomer) was the first discovered in 1984^3^ followed by its 5,7-epimer^4^, which was later renamed 8-epilegionaminic acid (8eLeg; L-*glycero*-D*-galacto* isomer) after the finding of legionaminic acid (Leg; D-*glycero*-D*-galacto* isomer) in 1994^5^. Pse and Leg were both named after the species in which the sugar was discovered. Later, a further non-2-ulosonic acid related to Leg was found and identified as the 4-epimer of Leg, 4-epilegionaminic acid (4eLeg; D-*glycero*-D*-talo* isomer)^6^, (Fig. 1A). In 2015, the 7,8-epimer of Leg (L-*glycero*-L-*altro* isomer; Fig. 1B) was found in the capsular polysaccharide (CPS) of a multiply antibiotic resistant *Acinetobacter baumannii* isolate, and named acinetaminic acid (Aci)^7^.

Each of these five naturally occurring 5,7-diamino-3,5,7,9-tetradeoxynon-2-ulosonic acids may have various acetyl or other acyl decorations on nitrogens 5 and 7 that can give rise to further structural diversity^2^, and the 5,7-di-*N*-acetylated derivative of acinetaminic acid, Aci5Ac7Ac, was produced by the *A. baumannii* isolate investigated^7^. The biosynthesis pathways for the 5,7-acetylated forms of Pse and Leg are inferred for *A. baumannii* based on experimental data from other species, and involve quite different steps^8^; Pse synthesis is mediated by Psa enzymes^9^, whereas Leg synthesis involves different Lga enzymes^10^. Though the synthesis pathway of 4eLeg has not been elucidated, genes for the synthesis of Leg were not found in the genome sequences of isolates in which this sugar was found, suggesting that 4eLeg is also synthesized by an alternate pathway. However, the gene clusters directing the synthesis of 8eLeg and Aci consist of genes for the synthesis of Leg plus additional genes for the modification of the Leg substrate, and possible pathways were predicted in a previous study^7^.

The discovery of Aci was facilitated by the discovery of novel genes in the KL12 capsule biosynthesis gene cluster located at the genomic K locus (KL). KL12 was found to contain a module of *lgaABCDEF* genes for the synthesis of CMP-Leg5Ac7Ac, with four novel genes adjacent to it that were predicted to convert this sugar to a novel monosaccharide. The K12 capsule was found to include Aci and the additional genes required for its synthesis were named *aciABCD* ^7^. Though these genes were found in more than one KL gene cluster, to date they have not been observed outside of the species, suggesting that the sugar is so far unique to *A. baumannii*.

Here, a variant of the *lgaABCDEF-aciABCD* gene cluster in which the *aciB* gene was replaced by a different gene, *aciE*, was discovered in a new *A. baumannii* genome sequence. The sugar content of the CPS produced by this strain was examined to determine the structure of the 5,7-diamino-3,5,7,9-tetradeoxynon-2-ulosonic acid present.

## Results

### KL73, a novel A. baumannii capsule biosynthesis gene cluster

**Figure 2:**
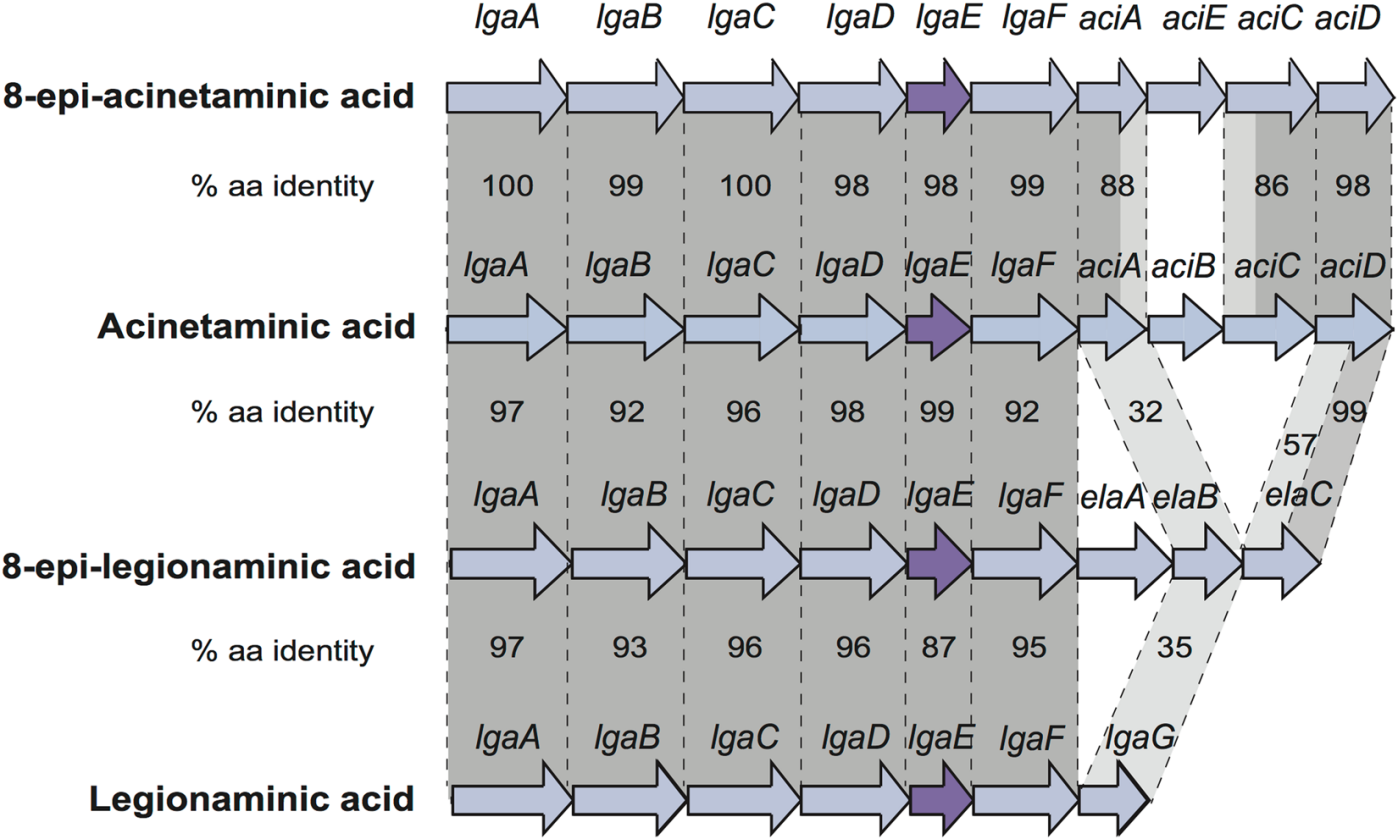
*A. baumannii* gene modules with *lga* biosynthesis genes. Gene modules are drawn to scale from GenBank accession numbers MF362178 (SGH 0703, 8-epiacinetaminic acid), NC_010400 (SDF, legionaminic acid), JN107991 (D36, acinetaminic acid) and JICJ01000028 (LAC-4, 8-epilegionaminic acid). Genes are represented by arrows showing the direction of transcription. Blue are nucleotide biosynthesis genes, and purple are acyl-/acetyl-transferase genes. Dark grey shading denotes >85% nucleotide sequence identity, and light grey is 70–85% nucleotide sequence identity. Amino acid sequence identities for proteins encoded by genes are shown.

The genome sequence of the multiply antibiotic resistant *A. baumannii* isolate SGH 0703 (DB24441 in GenBank accession number FPGS01000086.1) was found to carry a sugar synthesis module that differs from the module directing the synthesis of Aci by a single gene (Fig. 2). This module was in a novel capsule biosynthesis gene cluster at the K locus, designated KL73 (GenBank accession number MF362178). In the KL73 gene cluster one of the genes required for synthesis of CMP-Aci5Ac7Ac has been replaced by a different gene. KL73 shares 97.2% nucleotide sequence identity with KL12 (GenBank accession number JN107991) across the module including *lgaA-aciD*, differing only in a short 773 bp segment including the *aciB* gene (741 bp) and 16 bp at the 3’-end of *aciA*, such that the *aciA* sequence is 3 bp shorter in KL73 (Fig. 2). In KL73, the *aciB* gene is replaced with a gene, here designated *aciE*, which is predicted to encode a 265 aa short-chain dehydrogenase belonging to the adh_short_C2 (PF13561) protein family (Pfam). AciB (246 aa) was also predicted to be a short-chain dehydrogenase though it belongs to the adh_short (PF00106) family^7^. As the *aciC* and *aciD* genes appear to be intact and *aciA* is almost complete, it seemed likely that AciE is involved in the synthesis of a non-2-ulosonic acid related to 5,7-di-*N*-acetylacinetaminic acid.

The overall arrangement of genes in KL73 was not found in any other genome sequence available in the non-redundant and Whole Genome Shotgun (WGS) databases. However, it is closely related to those previously described for the *A. baumannii* KL12 and KL13 gene clusters which include the module for Aci5Ac7Ac synthesis^7^. Each of these gene clusters also includes a module of genes for the synthesis of UDP-L-Fuc*p*NAc (*fnlABC*) and of UDP-D-Fuc*p*NAc (*fnr1/gdr*)^8^. KL73 and KL13 contain the same *wzy* gene, encoding the polymerase responsible for linking K units together but KL12 includes a different *wzy* gene.

### CPS purification and chemical analyses

CPS from *A. baumannii* K73 was isolated after enzymatic treatment of the crude material present in the water layer of the hot water phenol extraction. Chemical analysis disclosed the occurrence of three different hexoses: D-galactose, L- and D-fucosamine. D-galactose was 4,6-linked and both L- and D-fucosamine were linked at position 3, as deduced by applying the methylation protocol for neutral sugars^11^. No terminal residue was detected, and it was assumed that the non-2-ulosonic acid foreseen by CPS gene cluster analysis was the lateral unit of the repeating unit of the polysaccharide.

### Isolation and NMR characterization of 5,7-diacetamido-3,5,7,9-tetradeoxy-D-*glycero*-L-*altro*-non-2-ulosonic acid (8eAci)

In order to determine the nature of the non-2-ulosonic acid missing in the chemical analysis, a pure CPS sample was hydrolyzed in mild conditions, and 6% AcOH (100 °C, 2 h) gave the best results. Chromatographic separation allowed the isolation of an acinetaminic acid-like monosaccharide later identified as its epimer at C-8 (Fig. 1C), while the remnant capsular polysaccharide partly depleted of the nonulosonic acid was eluted in the void volume of column and is not described in this work.

**Figure 3:**
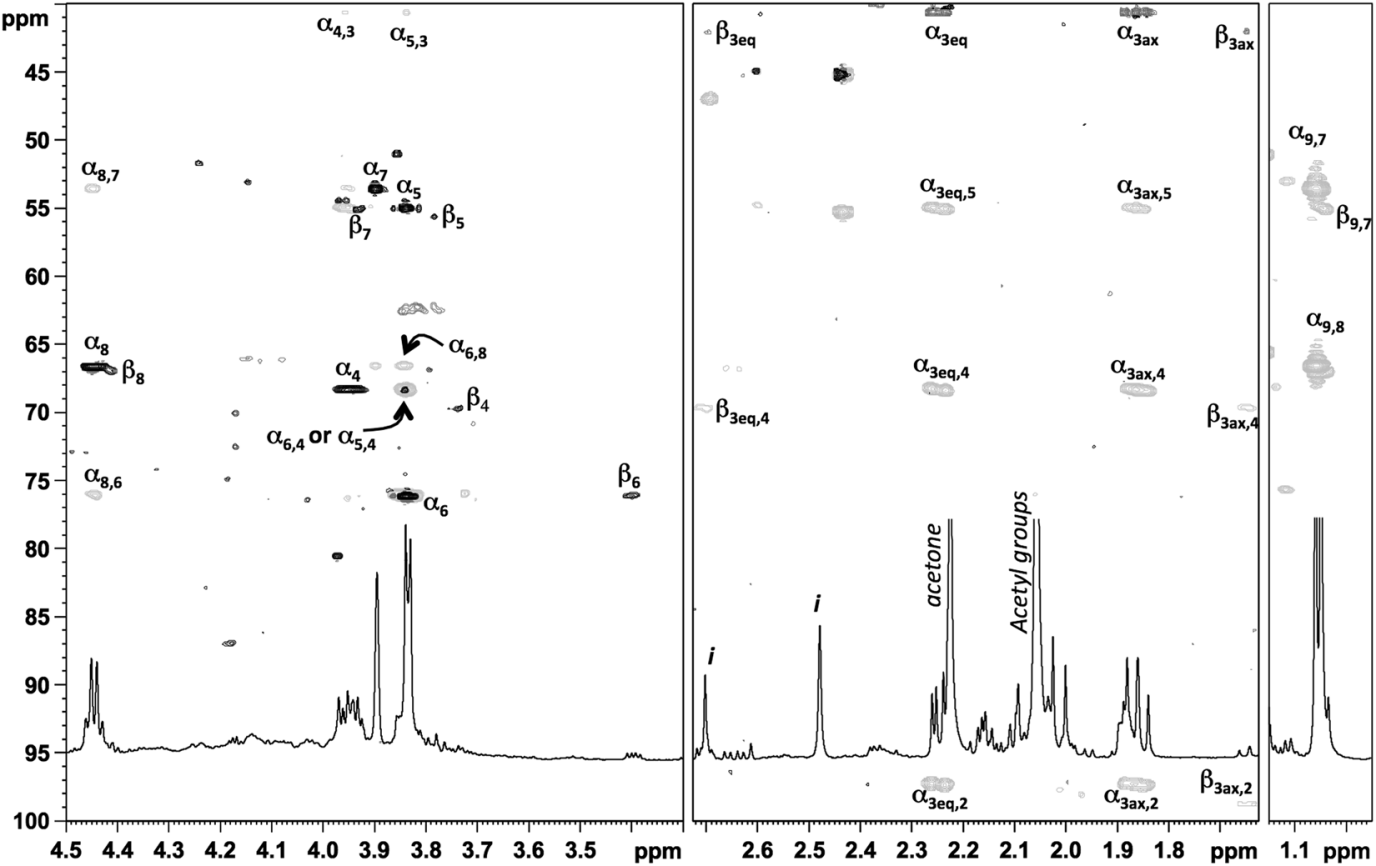
(^1^H 600 MHz, ^13^C 150 MHz, 25 °C) overlay of HSQC (black) and HMBC (grey) spectra measured for 8eAci monosaccharide (structure in Fig. 1C) isolated from the capsular polysaccharide from *A. baumannii* K73. “*i*” is impurity.

The proton spectrum of 8eAci displays an intense methyl signal at 1.05 ppm (Table 1, Fig. 3), another intense methyl signal at 2.05 ppm accounting for two acetyl groups, and a few proton signals at 4.5–3.4 ppm. Analysis of HSQC and HMBC spectra correlated the intense methyl signal at 1.05 ppm with two carbon signals at 66.5 and 53.5 ppm, assigned to C-8 and C-7 respectively. H-8 (4.45 ppm) had two long-range correlations, the first with C-7 and the second with a carbon at 76.1 ppm, assigned to C-6. The HSQC spectrum showed cross-peaks from two different protons at 3.84 ppm, one associated to C-6 and the other correlated to a carbon at 55.0 ppm. This last density was indeed attributed to C-5 as confirmed by the long range correlation between H-5 and a deoxy carbon atom at 40.5 ppm, namely C-3. The diastereotopic H-3 protons (2.24 and 1.86 ppm) had a long-range correlation with C-4 (68.3 ppm), C-2 (97.4 ppm) and C-1 (177.4 ppm). These attributions were confirmed by analyzing correlation spectroscopy (COSY) and total correlation spectroscopy (TOCSY) spectra, while proton-proton coupling constants were extracted from the proton spectrum (Table 1). Thus, this monosaccharide is 5,7-diacetamido-3,5,7,9-tetradeoxynon-2-ulosonic acid, α configured at the anomeric center and with the D-*glycero*-L-*talo* configuration of its five stereocenters (Fig. 1C) as established by comparison of its proton and carbon chemical shifts with those of the reference compound in the sodium salt form^12^. Indeed, the monosaccharide is the epimer at C-8 of the recently discovered acinetaminic acid^7^ and the name 8-epiacinetaminic acid (8eAci) is proposed.

**Table 1:** Proton (600 MHz) and carbon (150 MHz) chemical shifts of 8eAci (5,7-diacetamido-3,5,7,9-tetradeoxy-D-*glycero*-L-*altro*-non-2-ulosonic acid, *Fig. 1C) from the CPS of *A. baumannii* K73, measured at 25 °C.

	3_eq_; 3_ax_	4	5	6	7	8	9
α-8eAci	2.24;1.86	3.95	3.84	3.84	3.90	4.45	1.05
	40.5	68.3	55.0	76.1	53.5	66.5	20.3
	^2^ *J*_3eq,3ax_ = 13.2	ND	ND	^3^ *J*_6,7_ ≈ 0	^3^ *J*_7,8_ ≈ 0	^3^ *J*_8,9_ = 6.5	
	^3^ *J*_3eq,4_ = 4.9						
	^3^ *J*_3ax,4_ = 11.5						
β-8eAci	2.70;1.64	3.7	3.78	3.40	3.93	4.42	1.04
	41.9	69.7	55.5	76.1	54.9	66.8	20.3
	^2^ *J*_3eq,3ax_ = 12.2	^3^ *J*_4,5_ = 9.5	^3^ *J*_5,6_ = 9.5	^3^ *J*_6,7_ = 5.3	^3^ *J*_7,8_ ≈ 0	^3^ *J*_8,9_ = 6.0	
	^3^ *J*_3eq,4_ = 4.7						
	^3^ *J*_3ax,4_ = 12.2						

Spectra were calibrated with respect to internal acetone (^1^H: 2.225 ppm, ^13^C: 31.45 ppm). C-1 and C-2 values for α and β anomers were detected in the HMBC spectrum and were: 177.4 and 97.4 ppm, and 176.1 and 98.8 ppm, respectively. *For the chemical shift of the reference monosaccharide, refer to the sodium form of compound **51** in ref. 12.

Analysis of the NMR spectra by the same approach disclosed the presence of the minor β anomer of the residue; of note, in this case H-7 had a measurable coupling constant value with H-6 (^3^ *J*_6,7_ = 5.3 Hz) probably due to a different orientation of the exocyclic chain due to β (and not α) stereochemistry at the anomeric carbon. As for the α anomer, chemical shifts and coupling constant values measured for the β form of 8eAci agreed well with those published. Last, NMR spectra contained additional minor signals related to 8eAci, but their low intensity prevented the full assignment and therefore the understanding of the type of residue present.

## Discussion

This study describes the first report of the sixth naturally occurring 5,7-diacetamido-3,5,7,9-tetradeoxynon-2-ulosonic acid (D-*glycero*-L-*altro* isomer), which we have named 8-epiacinetaminic acid. Prior to this study, this sugar had never been found as a component of a natural polysaccharide, and had only been obtained previously by a synthetic route^12^. The new monosaccharide is the C8-epimer of Aci, and represents the final member of the C7/C8 epimers of this class, which includes Leg (C7-epimer of 8eAci), 8eLeg (C7/C8-epimer), and Aci (C8-epimer), to be found in nature. Our bioinformatics analysis suggests that the biosynthesis of each of these sugars proceeds via a pathway that first involves the production of CMP-Leg5Ac7Ac by enzymes encoded by *lgaABCDEF* genes. The additional genes present adjacent to this module determine which epimer the CMP-Leg5Ac7Ac substrate is converted to. The synthesis of Aci and of 8eAci appear to differ in only one enzyme, with AciE likely replacing the function of AciB to produce 8eAci. However, biochemical studies will be needed to elucidate both pathways.

Recently a further non-2-ulosonic acid, fusaminic acid, was found in *Fusobacterium nucleatum* strain 25586^13^ indicating that additional natural nonulosonic acids may remain to be discovered.

*A. baumannii* is a clinically significant human pathogen, with the majority of clinical isolates exhibiting resistance against multiple classes of antibiotics. The capsule has been shown to be involved in the virulence and survival of *A. baumannii*, as it provides protection for the cell presenting on the surface as a polysaccharide of a repeating oligosaccharide known as a K unit. Of more than 100 *A. baumannii* capsule gene clusters discovered to date, ~30% (32) contain genes for one of the known non-2-ulosonic acids, indicating the potential importance of these sugars. However, only one isolate, DB24441, carries genes for the synthesis 8eAci. Though non-2-ulosonic acids have been implicated in virulence in other Gram-negative species, studies are still required to determine their role in the virulence of *A. baumannii*.

## Methods

### Bacterial strain

*A. baumannii* SGH 0703 (DB24441) is a multiply antibiotic resistant clinical isolate recovered at the Singapore General Hospital in 2007. SGH 0703 belongs to Global Clone 2 (GC2; ST2 in the Pasteur MLST scheme) and to ST1165 (a single locus variant of ST208) in the Oxford scheme. K73 was grown in Luria broth (LB) for 16 hrs at 37 °C and cells were collected by centrifugation (5000 *g*) at 4 °C for 30 mins. The cell pellet was resuspended in 0.9% saline, autoclaved, and then freeze-dried prior to capsule extraction, yielding 9 g of dried cell mass.

### CPS extraction and chemical analysis

*A. baumannii* K73 cells (5.0 g) were extracted sequentially with phenol-chloroform-petroleum (PCP; 2:8:5 v/v/v)^14^ and hot water/phenol method^15^ to isolate the lipooligosaccharide (LOS) and CPS. As expected, LOS was found in the PCP cells extract, while CPS (yield 11.3% g_CPS_/g_cells_) was in the water layer; LOS was not further studied and analysis proceeded on CPS.

The capsule was purified from nucleic acid and proteins by enzymatic treatment as reported^11^ and recovered in 22% yield. Monosaccharide compositional analysis (acetylated methylglycosides), determination of the substitution pattern and absolute configuration (octyl glycosides), were performed as reported^11^.

GC-MS analyses were performed with an Agilent instrument (GC instrument Agilent 6850 coupled to MS Agilent 5973), equipped with a SPB-5 capillary column (Supelco, 30 m × 0.25 i.d., flow rate, 0.8 mL min^−1^) and He as carrier gas. Electron impact mass spectra were recorded with an ionization energy of 70 eV and an ionizing current of 0.2 m A. The temperature program used for all analyses was: 150 °C for 5 min, 150 → 280 °C at 3 °C/min.

### CPS from mild acid hydrolysis

Purified CPS (5 mg) was hydrolyzed in aq. 6% AcOH (100°C, 2 h), lyophilized and purified by size exclusion liquid chromatography run in water on a BioGel P-10 column (Bio-rad, flow = 10 ml/h, d = 1.5 cm, h = 110 cm), the eluate was monitored by an on-line refractive index detector (Knauer K-2301), and fractions were pooled according to the chromatographic profile recorded. CPS related material (4.0 mg), named dCPS, was recovered in the void volume of the column, while 8eAci (0.5 mg) monosaccharide was eluted later. Attempts to optimize the removal of the nonulosonic acids (aq. AcOH 1%, at 100°C for 2 and 4 h, aq. AcOH 6% at 100°C for 4 hrs) gave unsatisfactory results. A consistent amount of 8eAci was always found on the CPS, even when hydrolyzed CPS was treated for a second time using the same conditions. On the other hand, we observed that prolonging acid treatment decreased the recovery yields of 8eAci, and we could never identify the nature of the byproducts related to this residue. Indeed, aq. 6% AcOH (100°C, 2 h) are the best we could develop in the course of this study.

### NMR spectroscopy

NMR experiments were performed using a Bruker DRX at 600 MHz, equipped with a cryogenic probe, routine spectra were acquired at 25 °C and calibrated on acetone (^1^H 2.225 ppm, ^13^C 31.45 ppm) used as internal standard. Two-dimensional spectra (DQF-COSY, TOCSY, gradient selected heteronuclear single quantum correlation (gHSQC), and gradient selected heteronuclear multiple bond correlation (gHMBC)) were measured using the standard Bruker software (Topspin 2.1). For the homonuclear experiments, 512 FIDs of 2048 complex data points were collected, with 24 scans per FID, a mixing time of 100 ms was applied for the TOCSY spectrum. Processing of Bruker data and spectra analysis was performed with Bruker TopSpin 3 program.

### Bioinformatics

The genome isolated from *A. baumannii* DB24441 was sequenced as described previously^16^, and deposited in GenBank under accession number FPGS01000086.1. The gene cluster at the K locus was characterised and annotated as described previously^8^.

### Data availability

The annotations for the KL73 sequence generated during and/or analysed during the current study are available in the GenBank repository, under accession number MF362178.
